# Effects of testosterone replacement on serotonin levels in the prostate and plasma in a murine model of hypogonadism

**DOI:** 10.1038/s41598-020-71718-z

**Published:** 2020-09-07

**Authors:** Paulo Mota, João Barbosa-Martins, Rute S. Moura, Estêvão Lima, Alice Miranda, Jorge Correia-Pinto, Emanuel Carvalho-Dias

**Affiliations:** 1 School of Medicine, Life and Health Sciences Research Institute (ICVS), University of Minho, 4710-057 Braga, Portugal; 2 ICVS/3B’s - PT Government Associate Laboratory, 4710-057 Braga/Guimarães, Portugal; 3 Department of Urology, Hospital of Braga, E.P.E, Braga, Portugal; 4 Department of Pediatric Surgery, Hospital de Braga, E.P.E., Braga, Portugal

**Keywords:** Prostate, Benign prostatic hyperplasia

## Abstract

Benign prostate hyperplasia is a dysfunctional disease with an elevated prevalence. Despite the accepted impact of aging and testosterone (TES) in its pathophysiology, its aetiology remains unknown. Recent studies described that serotonin (5-HT) inhibits benign prostate growth through the modulation of the androgen receptor, in the presence of TES. Accordingly, this work aimed to determine the impact of castration and TES replacement in plasmatic and prostatic 5-HT regulation. C57BL/6 mice were submitted to surgical castration and divided into three groups, continually exposed to either vehicle or different TES doses for 14 days. Plasmatic 5-HT concentration was measured before and after castration, and after TES reintroduction. Finally, total prostatic weight and intra-prostatic 5-HT were determined in the different groups. Our results demonstrate that mice prostate exhibits high 5-HT tissue levels and that intra-prostatic total 5-HT was independent of castration or TES reintroduction, in all studied groups. Also, 5-HT plasmatic concentration significantly increased after castration and then normalized after TES administration. Our findings revealed that mice prostate has a high 5-HT content and that total prostatic 5-HT levels do not depend on androgens’ action. On the other hand, castration induced a significant increase in plasmatic 5-HT concentration, raising the hypothesis that androgens might be regulating the production of extra-prostatic 5-HT.

## Introduction

Benign prostate hyperplasia (BPH) is the main cause of non-neurogenic lower urinary tract symptoms (LUTS) in men^1,2^. Its prevalence rises after the fourth decade of life and, in men over 80 years old, is around 90%^2^. Despite being one of the most common human diseases, the aetiology of BPH is still unknown. Nonetheless, it is consensual that aging and the presence of testosterone (TES) are important risk factors for BPH^3^.

Several reports have documented that, in aging males, plasmatic TES is decreased^4,5^; also, intraprostatic levels of TES or dihydrotestosterone are not increased in aged men with BPH^6^. Additionally, if hypogonadic or even normogonadic men are treated with supra-physiologic TES concentrations, their prostate glands do not increase in size^7^. These findings were, in part, responsible for the development of the saturation hypothesis for prostate growth that claims androgens are crucial for prostatic growth (benign or malignant); however, the concentration of TES necessary for saturation of all prostatic androgen receptors (AR) is near the castration range, thus concluding that androgens are not the cause of BPH nor prostate cancer^7^.

Serotonin (5-HT, 5-hydroxytryptamine) is a biogenic monoamine that has numerous functions in humans. Despite its well-known role in the central nervous system, the vast majority (95%) of total body 5-HT is found at the periphery^8^. Importantly, in the prostate, 5-HT is one of the most abundant neuroendocrine factors present in neuroendocrine cells (NEC)^9^. It has been reported, in a large humans study, that LUTS were associated with BPH and, at the same time, with a decrease in plasmatic 5-HT concentration^10^. Several reports have also demonstrated that NEC and 5-HT are significantly decreased in BPH when compared to normal prostate^11–13^. Recently, our group showed that 5-HT is a strong inhibitor of benign prostate growth through down-regulation of the AR. Additionally, we demonstrated that mice depleted from peripheral 5-HT had a higher prostatic mass, and an up-regulation of the AR^14^. This data suggests that depletion of 5-HT in the prostate transition zone could be the etiologic factor responsible for the development and progression of BPH through neuroendocrine dysregulation. Still, the physiologic interaction between TES and 5-HT is unknown. Accordingly, in this study, we asked whether androgens (the main stimulator of prostate growth) could regulate 5-HT production (the inhibitor of prostate growth) since 5-HT appears to counteract the stimulatory effect of androgens over prostate growth^14^. Therefore, in this study, we evaluated the effect of TES over plasmatic and prostatic 5-HT concentration using a murine model of hypogonadism.

## Materials and methods

### Ethics and animal work

**Figure 1:**
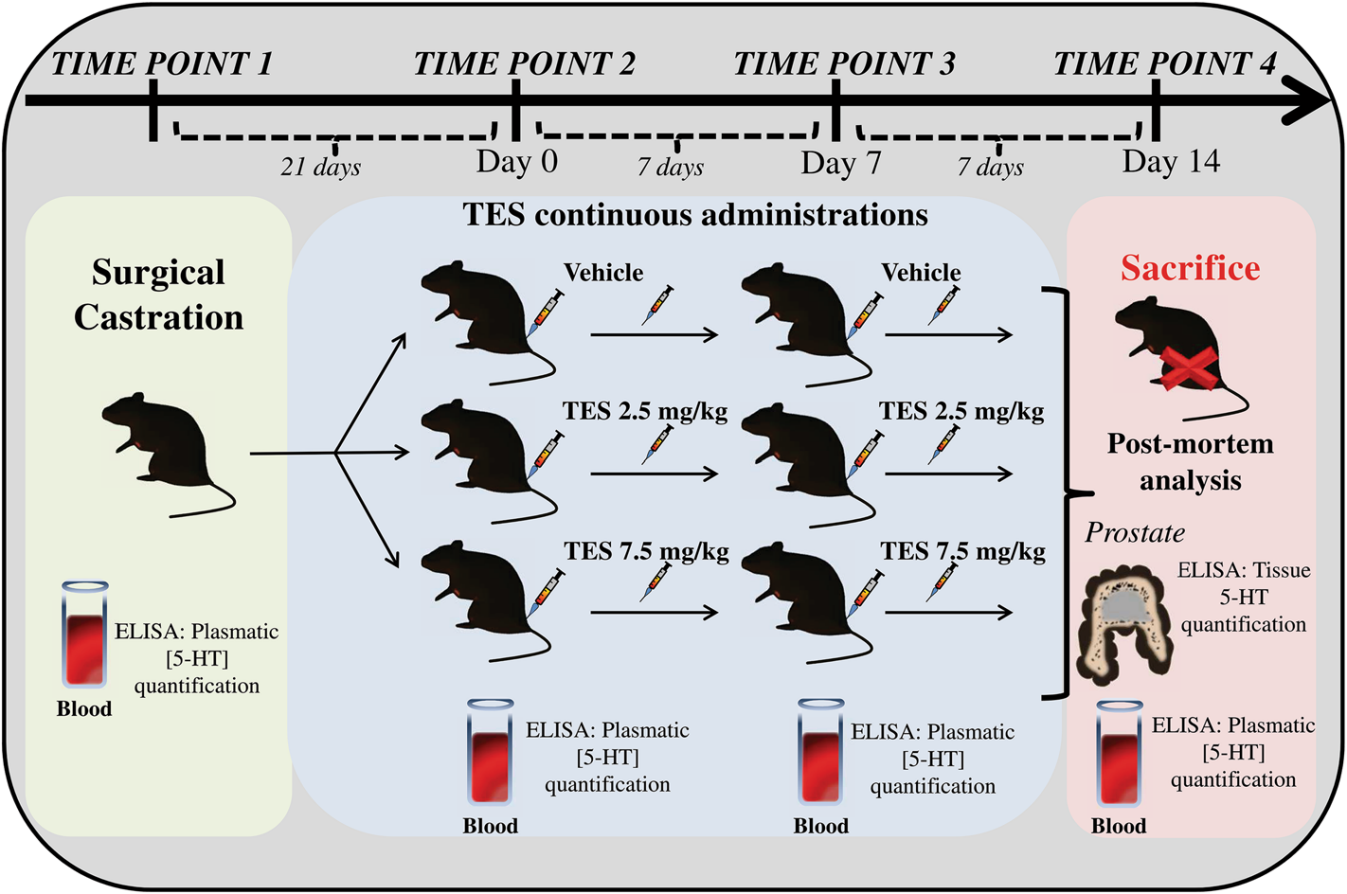
Schematic representation of the experimental design. Timeline for surgical castration, vehicle, TES 2.5 mg/kg or TES 7.5 mg/kg continuous administration and sacrifice of C57BL/6 mice. Plasma and tissue analysis at each time point, is also indicated. 5-HT, serotonin; TES, testosterone. (Fig. 1 was constructed by Barbosa-Martins, J.).

Mice were maintained following the guidelines of “Guide to the Care and Use of Experimental Animals” National Academy of Science and the EU Directive 2010/63/EU. This research project was approved by the Animal Ethics Committee of the University of Minho (SECVS 003/2016) and by the National Competent Authority for Animal Protection (DGAV 0421/000/000/2016). Adult male C57BL/6 mice (Charles River, Spain), 5/6 months old, mean weight of 28 g, were housed in an animal facility with controlled temperature, humidity and artificial light/dark cycle (temperature 20–24 °C, humidity 55 ± 10% and 12:12 h light/dark cycle). Irradiated food and autoclaved water were provided ad libitum. Researchers performing the experiments were blinded to the experimental and control groups during all testing. All experiments were performed during the light phase and at the same daytime period. The experimental study design is represented in Fig. 1.

### Animal castration

Male mice were anesthetized with intraperitoneal (IP) 75 mg/kg of ketamine (Imalgene, Merial, France) and 1 mg/kg of medetomidine (DorbeneVet, SYVA, Spain). Analgesia was provided by subcutaneous administration (SC) of 0.05 mg/kg of buprenorphine (Bupaq, Richter Pharma AG, Austria). Animals were then surgically prepared, and orchidectomies were performed as previously described^15^. Animals were monitored daily for signs of infection, pain, and discomfort.

### TES administration

TES propionate (Sigma-Aldrich; St. Louis, Missouri, USA) was daily prepared in sesame oil vehicle (Sigma-Aldrich). Twenty-one days after castration, male mice were randomly assigned to three experimental groups: Group 1—Control group receiving sesame oil (vehicle) (n = 6); Group 2—mice receiving 2.5 mg/kg TES (n = 7); Group 3—mice receiving 7.5 mg/kg TES (n = 7). IP injections were administrated daily for 14 days. TES doses, vehicle, and time of supplementation were chosen considering previous reports^16–18^.

In order to measure 5-HT plasmatic concentration, blood was collected on tail vein on the day of castration (time point 1), on the first day of injection of sesame oil or TES at 2.5 or 7.5 mg/Kg (time point 2), after 7 days of injections of sesame oil or TES at 2.5 or 7.5 mg/Kg (time point 3) and at the end of 14 days of injections of sesame oil or TES at 2.5 or 7.5 mg/Kg (time point 4). Body weight was assessed at each time point, and animals were monitored daily for signs of discomfort. Twenty-four hours after the last injection, animals were anesthetized by exposure to isoflurane 3% (IsoFlo, Zoetis, Portugal), and then decapitated for total blood exsanguination. Prostatic tissue (all lobes combined) was microdissected, removed from fat and other urogenital tissues, under a dissection microscope (Olympus SZX16, Japan), as previously described^14^, and photographed. Total prostate (all right and left lobes together) was weighted immediately after dissection and stored at  − 80 °C. The prostatic index was calculated using the formula: prostate wet weight/mouse weight.

### Plasmatic 5-HT enzyme-linked immunosorbent assay

At each time point, blood was collected using capillary tubes coated with sodium heparin. Samples were centrifuged at room temperature, for 10 min at 200 g, to obtain platelet-rich plasma. Then, an aliquot of the platelet-rich plasma was centrifuged at 4500 g for 10 min at 4 °C to obtain platelet-free plasma, and samples were frozen at  − 80 °C. Mice plasmatic 5-HT concentration was measured using the commercial kit, IBL Serotonin ELISA (RE59121, IBL, Hamburg, Germany), as previously described^19^. All procedures were performed according to the manufacturer’s instructions.

### Prostatic 5-HT enzyme-linked immunosorbent assay

The prostatic 5-HT content was measured using the commercial IBL Serotonin ELISA kit (RE59121). Briefly, prostatic tissue was homogenized on ice, using a pellet pestle cordless motor (Kontes Glass; Vineland, NJ, USA) and a lysis buffer (CelLytic MT; Sigma-Aldrich) that contained a protease inhibitor cocktail (Sigma-Aldrich), as previously described for other tissues^20,21^. Subsequently, the tissue was further homogenized by sonication (Vibra Cell, SONICS; Newtown, Connecticut, USA) at an amplitude of 30 for 30 s at pulse of 2, adapted from previous work^12^. The homogenate was centrifuged at 15,300 g for 15 min, the supernatant collected and stored at  − 80 °C. Prostatic 5-HT content was measured in the supernatants using the commercial IBL Serotonin ELISA kit (RE59121). All procedures were performed according to the manufacturer’s instructions.

### Statistical analysis

Statistical analysis was performed using SPSS software version 22 (SPSS; Chicago, IL, USA). Graphs were made using GraphPad Prism version 6 (San Diego, California, USA). The normality of distribution was tested using the Shapiro–Wilk test and, if indicated, kurtosis and skewness were evaluated. Statistical analysis used either Student’s t-test or ANOVA and Bonferroni post-tests or Pearson’s bivariate correlation, when appropriate. *p* < 0.05 was considered significant. All experimental data are presented as mean ± standard error (s.e.m.).

## Results

### Total prostatic 5-HT is independent of androgen status

**Figure 2:**
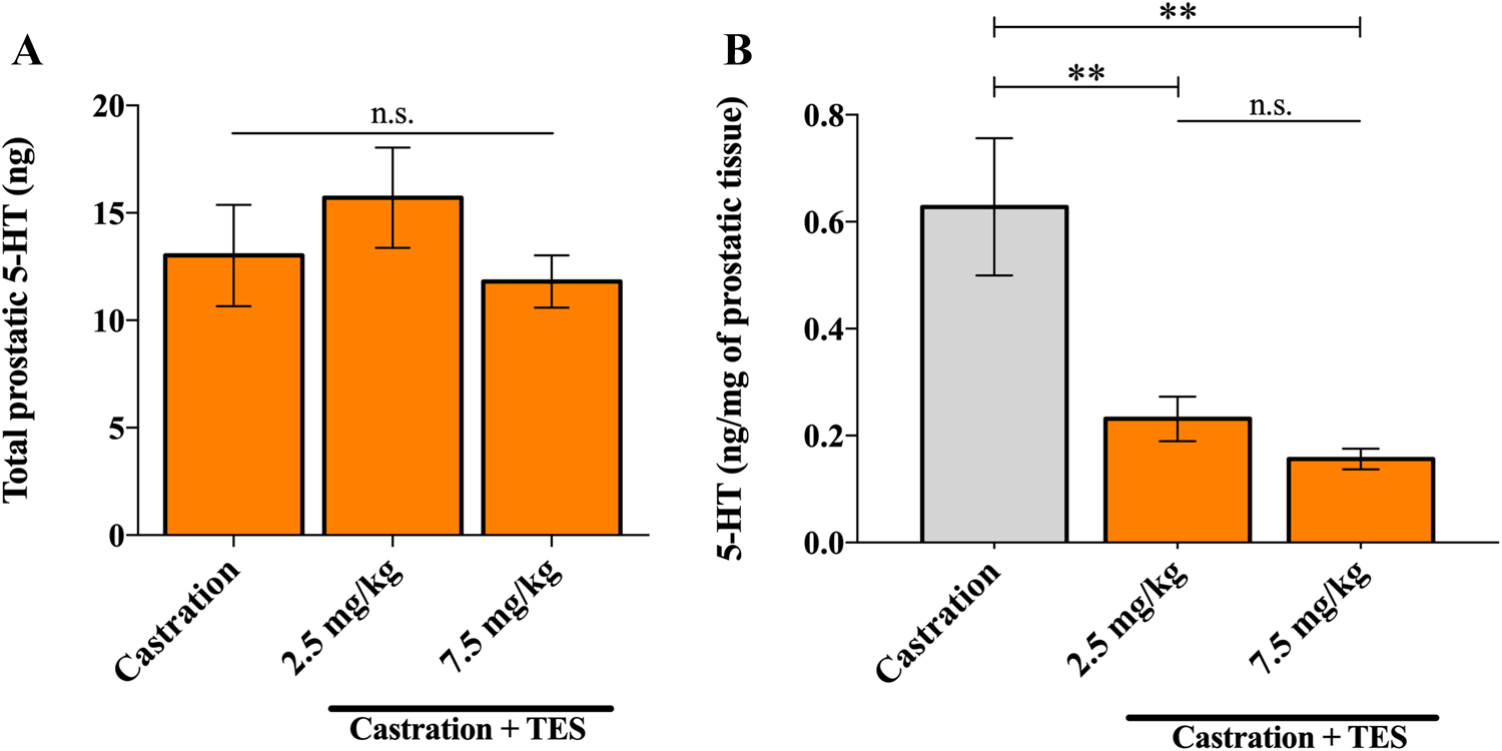
Effect of castration or TES administration in mice prostatic 5-HT (**A**) Quantification of total 5-HT in prostatic tissue by ELISA assay, after 14 days of continuous administration of vehicle (n = 5), 2.5 mg/kg (n = 5) or 7.5 mg/kg of TES (n = 7). (**B**) Absolute levels of 5-HT were normalized to the corresponding prostate weight. Data are presented as mean ± SEM. A one-way ANOVA, followed by a Bonferroni post-hoc, was used to compare data. n.s.—non-significant; **p = 0.006, 2.5 mg/kg vs. castration; **p = 0.001, 7.5 mg/kg versus castration.

Castration and TES reestablishment did not induce significant differences in total prostatic 5-HT levels between groups (*p* = 0.354; Fig. 2A). However, after normalization to prostatic mass, significant differences were observed (*p* = 0.001). The castrated group had significantly higher prostatic 5-HT concentration than the 2.5 mg/kg group (*p* = 0.006; Fig. 2B) or the 7.5 mg/kg group (*p* = 0.001; Fig. 2B). No significant differences were found between the two TES receiving groups (*p* = 1.00; Fig. 2B).

### Castration increases plasmatic 5-HT concentration whereas TES replacement decreases plasmatic 5-HT concentration

**Figure 3:**
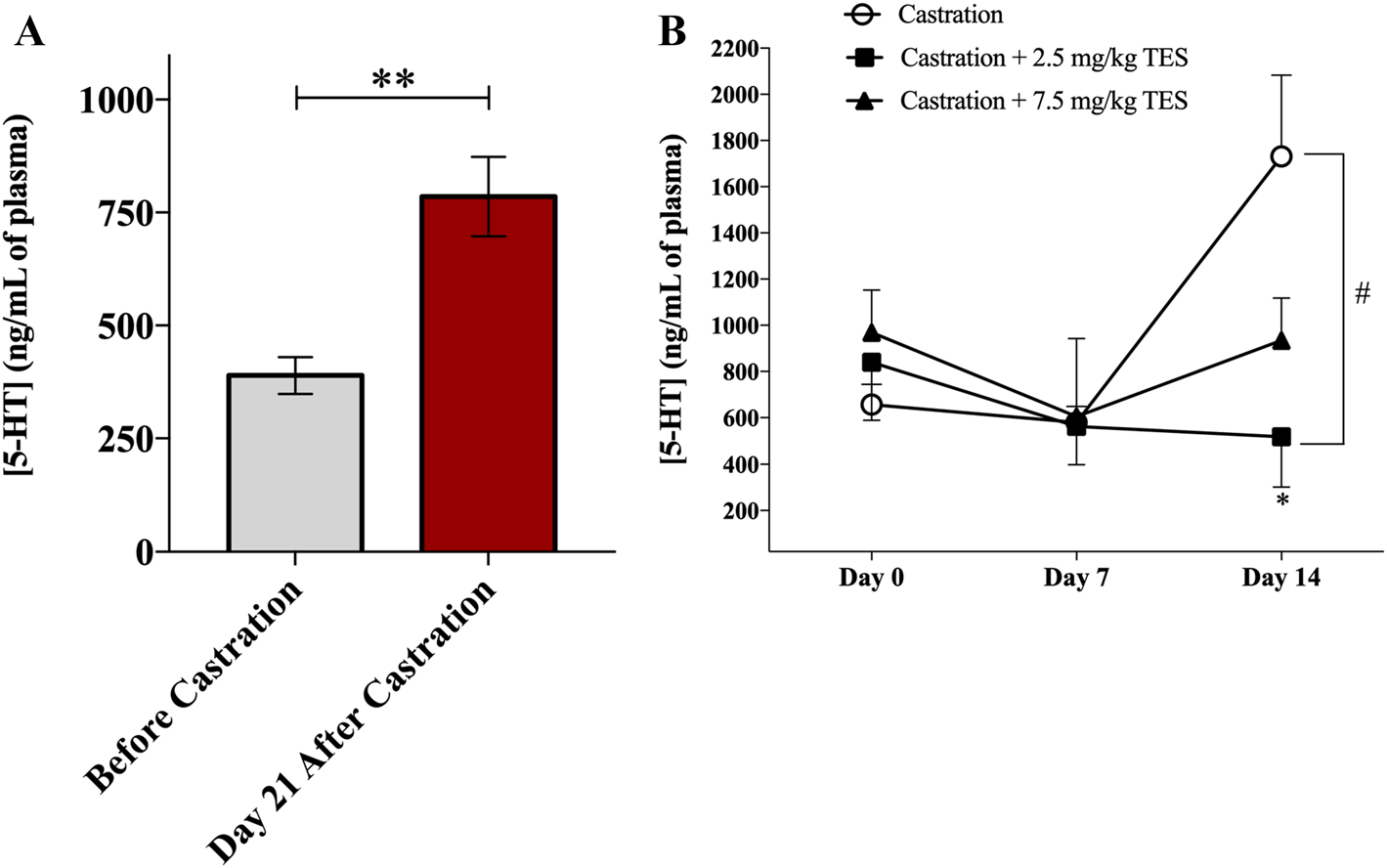
Effect of castration and TES re-introduction in plasmatic 5-HT concentration. (**A**) Quantification of plasmatic 5-HT concentration by ELISA assay in mice before and after 21 days of castration (n = 17). **p = 0.001. A paired samples T-test was used to compare each time point. (**B**) Quantification of 5-HT plasmatic concentration before, and after 7 and 14 days of continuous administration of vehicle (n = 6), 2.5 mg/kg (n = 4) or 7.5 mg/kg (n = 5) of TES, by ELISA assay. Data are presented as mean ± SEM. A one-way ANOVA, followed by a Bonferroni *post-hoc* test was used to compare Day 14 5-HT concentrations between groups. A mixed factorial ANOVA, followed by a Bonferroni *post-hoc* test was used to compare each time point and groups. *p = 0.043, Day 14 versus Day 0 to castration + 2.5 mg/kg group; #*p* = 0.038, castration versus castration + 2.5 mg/kg at Day 14.

Castration significantly induced a twofold increase in plasmatic 5-HT concentration (*p* = 0.001; Fig. 3A). This tendency was even more pronounced after 35 days of castration, particularly from day 7 to day 14, as concentration increased about threefold (579.5 ± 68.7 vs. 1731.3 ± 351.8 ng/mL; p = 0.073; Fig. 3B). Conversely, plasmatic 5-HT concentration was not affected by TES injections as there were no significant differences between the two doses at any time point (*p* = 1.00; Fig. 3B).

**Figure 4:**
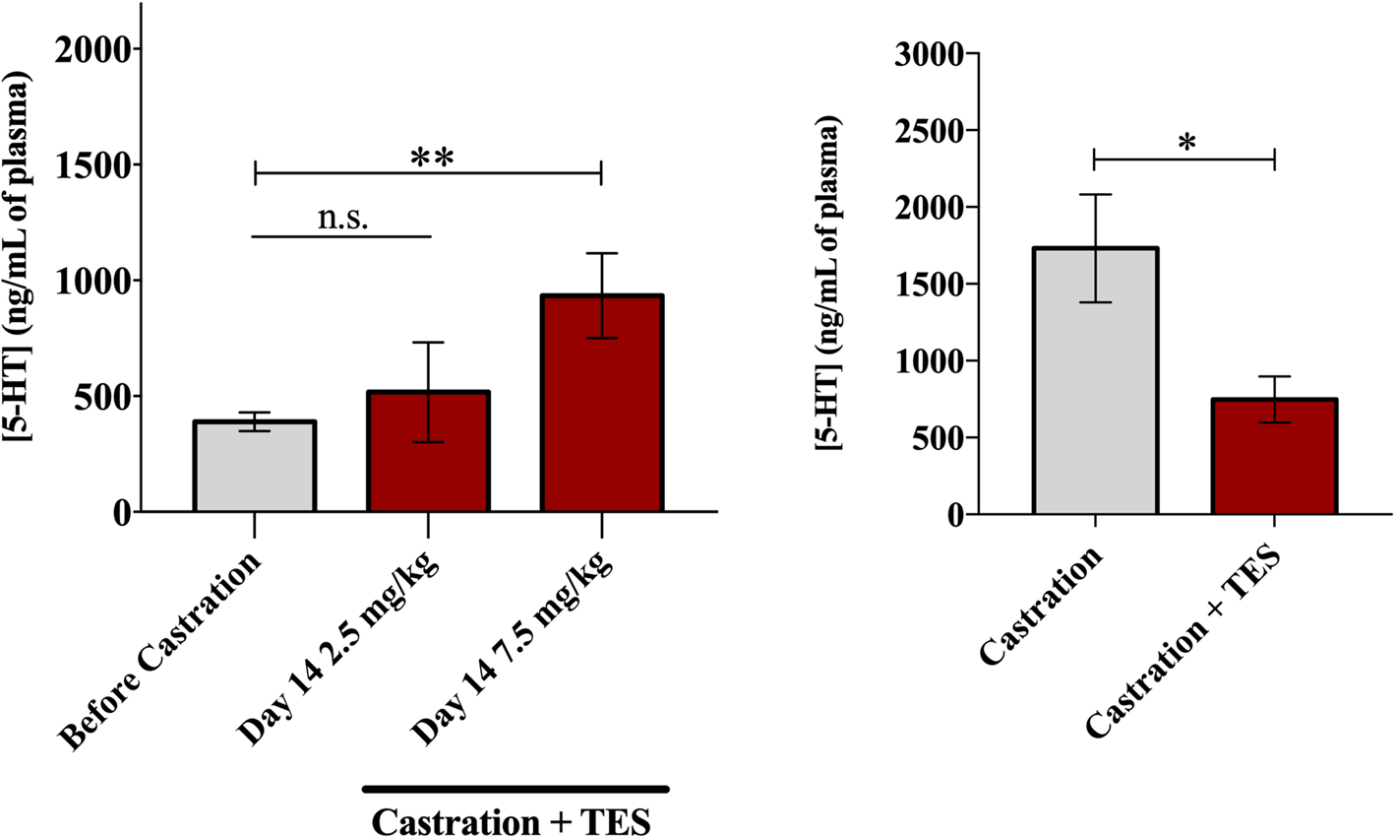
5-HT plasmatic concentration in experimental groups. (**A**) Quantification of plasmatic 5-HT concentration by ELISA assay in mice before castration (n = 17) and after 14 days of TES 2.5 mg/kg (n = 4) and TES 7.5 mg/kg (n = 5) re-introduction. Data are presented as mean ± SEM. A one-way ANOVA, followed by a Bonferroni post-hoc was used to compare data. n.s.—non-significant; **p = 0.002. (**B**) Comparison between 5-HT concentration levels of control (n = 6) and both TES-receiving groups (n = 9), at Day 14. Data are presented as mean ± SEM. An independent samples T-test was used to compare each data. *p = 0.012.

After TES replacement, the 5-HT plasmatic concentration decreased to similar levels before castration; nonetheless, in mice receiving 7.5 mg/Kg of TES, 5-HT plasmatic levels were not completely restored to levels before castration (Fig. 4A). This observation was also evident when mice after 35 days of castration were compared with both TES-receiving groups. In this case, a significant decrease in plasmatic 5-HT concentration was observed in mice treated with TES comparatively to castrated animals (*p* = 0.012; Fig. 4B).

### Plasmatic 5-HT correlates with prostatic 5-HT

**Figure 5:**
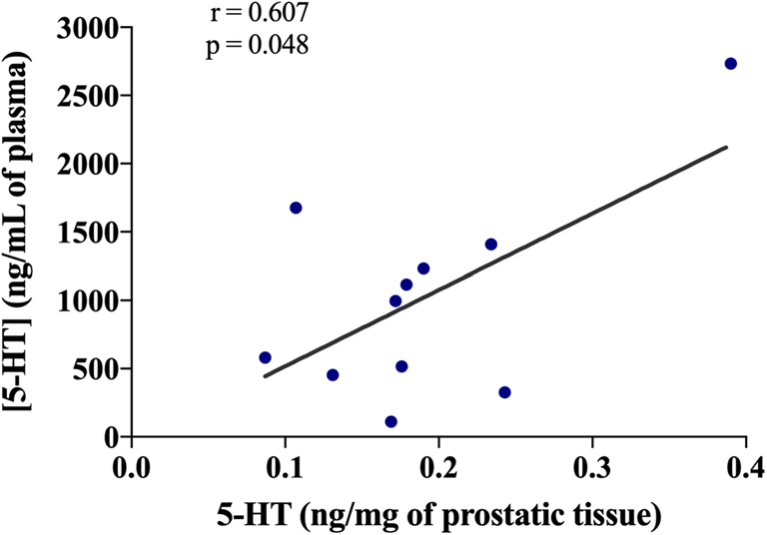
Correlation between plasmatic and prostatic 5-HT, to similar prostatic masses. A scatter diagram and Pearson’s bivariate correlation was used to compare plasmatic and prostatic 5-HT levels of TES group at day 14 (n = 11). r = 0.607; *p* = 0.048.

Plasmatic and prostatic 5-HT concentration was analysed in all animals that received TES re-administration. Animals with higher plasmatic 5-HT concentration also presented a higher prostatic 5-HT concentration (*p* = 0.048; Fig. 5).

### Prostatic size is not dependent on TES concentration re-administration

**Figure 6:**
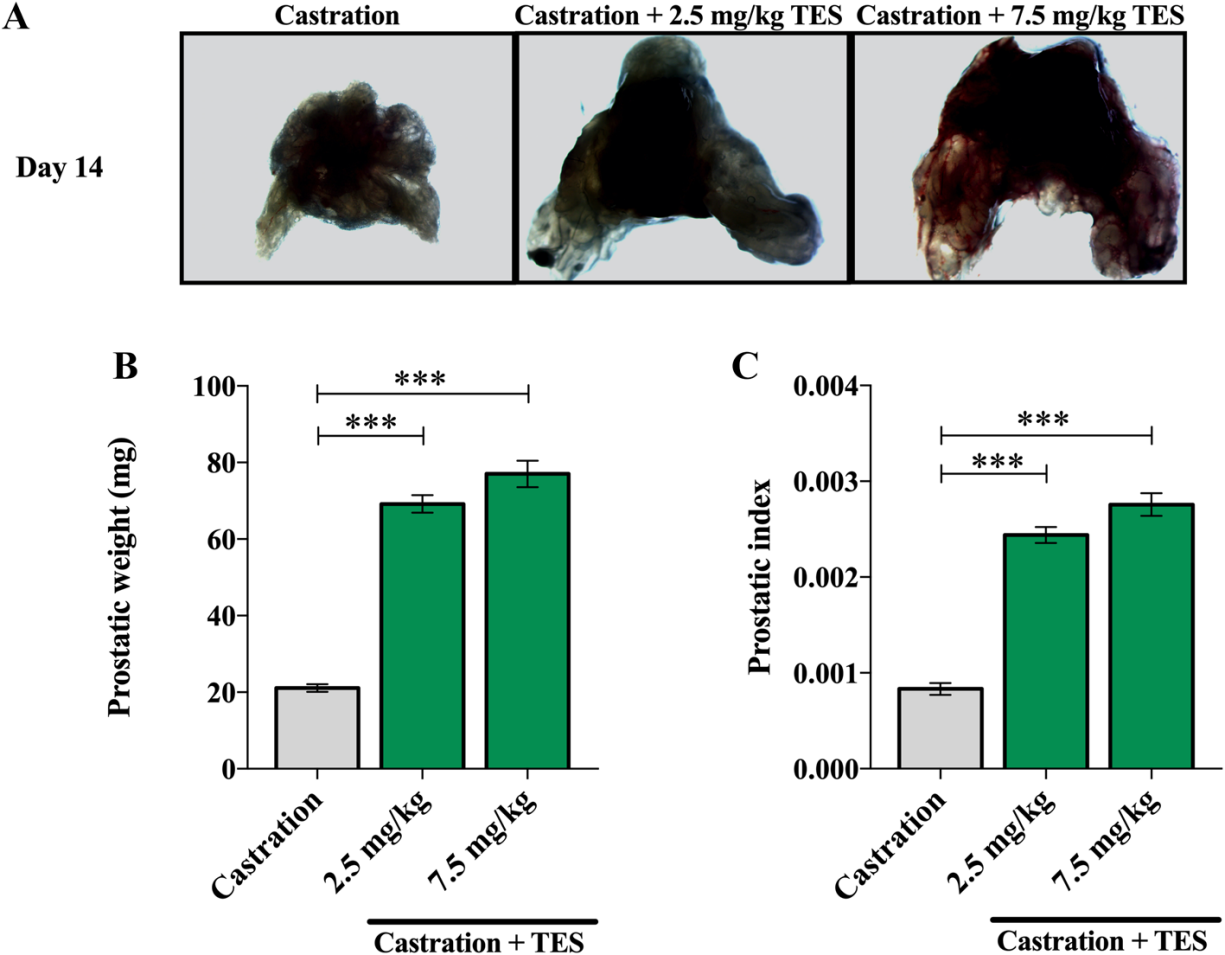
Effect of TES in mice prostatic size and weight. (**A**) Representative examples of mice prostatic tissue for the three experimental groups. (**B**, **C**) Dissected prostate weight (mg) and prostatic index for control (n = 5), 2.5 mg/kg (n = 5) and 7.5 mg/kg (n = 7) of TES receiving groups. Data are presented as mean ± SEM. A one-way ANOVA, followed by a Bonferroni post-hoc, was used to compare each data. ***p < 0.001. (Photos of (**A**) were taken by Mota, P. and Carvalho-Dias, E.).

Castration or exposure to different TES dosages led to significant differences in mice prostates (Fig. 6A). As expected, prostatic weight was significantly higher in the TES receiving groups than in the castration group (*p* < 0.0001; Fig. 6B). Nevertheless, there were no significant differences between the two TES receiving groups (*p* = 0.183). Similarly to prostatic weight, the prostatic index was significantly increased in the TES receiving groups compared to the castration group (*p* < 0.0001; Fig. 6C). Also, in this case, there were no significant differences between TES receiving groups (*p* = 0.119).

### Body weight is reduced by castration and restored by TES

**Figure 7:**
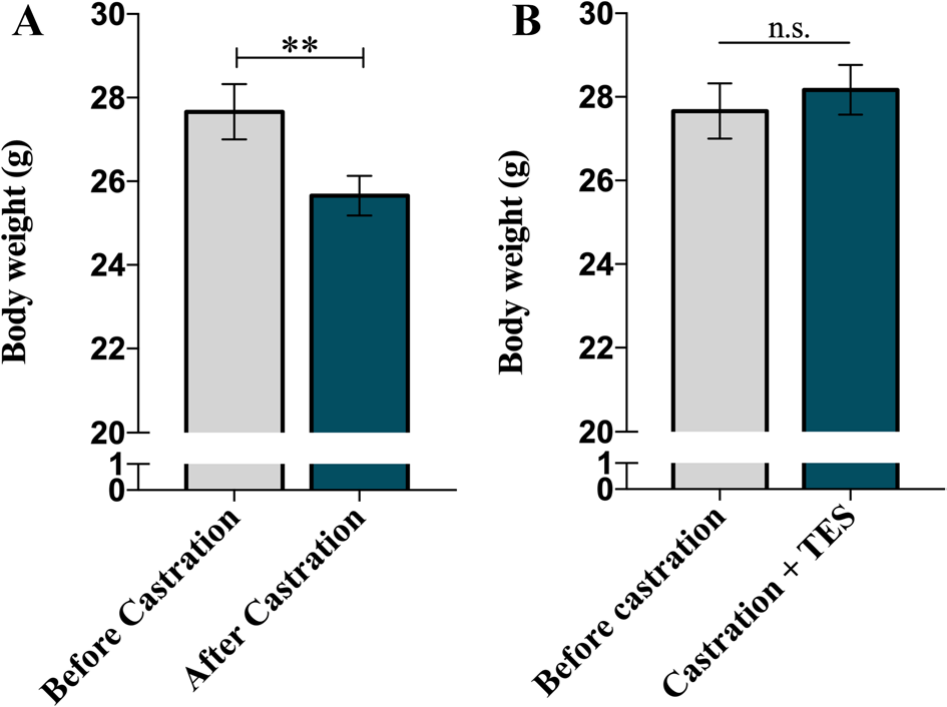
TES effect on mice body weight. (**A**) Representation of total body weight before and after 21 days of surgery (n = 20). Data are presented as mean ± SEM. A paired samples T-test was used to compare each time point. **p = 0.005. (**B**) Representation of total body weight before castration (n = 20) and after 14 days of continuous administration in both TES-receiving groups (n = 12). Data are presented as mean ± SEM. An independent samples T-test was used to compare each data. n.s.—non-significant.

Castration significantly reduced mice body weight (*p* = 0.005; Fig. 7A). TES re-administration completely restored animal weight to levels before castration (*p* = 0.607; Fig. 7B).

## Discussion

Considering the recognized effect that 5-HT seems to have on prostatic growth, in the present study, we aimed to evaluate the impact of androgen modulation over the main neuroendocrine prostatic factor, namely 5-HT. For this purpose, we used a hypogonadism mouse model to have full control of TES levels and then accurately assess its impact on prostate physiology. We have not, however, perform the studies under basal conditions. The castration model used in this study is very well characterized in the literature and it has been extensively shown that surgical castration efficiently reduces plasma TES levels post-surgery^22,23^. For this reason, we did not assess the plasma TES levels in our study. Nonetheless, the reduction observed on the size of the prostate gland after castration, along with the decrease of animal’s body weight, indirectly reflects the loss of TES activity of over the prostate gland thus, implicitly validating the model. Androgen deprivation is known to impact specific regions of prostate tissue, namely, the acinar epithelium, the number of mast cells and collagen fibers^24^.

The presence of 5-HT receptors in the normal prostate tissue has been recently characterized, revealing that 5-Htr1a is predominantly expressed in the epithelium, whereas 5-Htr1b is expressed in both epithelium and stroma^14^. Conversely, to the best of our knowledge, this is the first time that the total amount of 5-HT in mice prostate is quantified, and, surprisingly, the prostatic concentration of 5-HT is very high. In all TES supplemented animals, prostatic 5-HT concentration (0.188 ng/mg) is slightly inferior to the values found in the frontal cortex (0.8 ng/mg)^25^, raphe (1.7 ng/mg) or spinal cord (1.2 ng/mg)^26^, which are the areas where 5-HT is more abundant in the nervous system. As expected, the prostatic concentration of 5-HT is considerably lower than in the duodenum (25 ng/mg), which is the primary source of peripheral 5-HT^27^. The high levels of prostatic 5-HT are in agreement with our previous results, demonstrating that 5-HT is a potent negative regulator of benign prostatic growth.

Furthermore, prostatic 5-HT concentration does not seem to be related to androgens plasmatic concentration since it is not dependent on castration or TES administration. Our data, based on a functional approach, is in agreement with previous reports suggesting that prostatic NEC secretion is independent, directly or indirectly, of androgens’ action. Prior studies have only demonstrated that castration or/and TES administrations do not alter NEC population nor their morphologic features^28,29^. Collectively, these findings led us to speculate that 5-HT production by NEC may be constant and induce a continuous inhibitory effect over prostatic growth independently of androgens.

Our data demonstrated that plasmatic 5-HT concentration continuously increased after castration and decreased after TES re-administration, showing that, contrary to what was observed locally in mice prostate, androgens seem to determine plasmatic 5-HT concentration. Our data is in accordance with a previous report that revealed that plasmatic 5-HT concentration decreases after TES administration; however, and contrary to that report, we also observed that castration increases plasmatic 5-HT concentration^18^. The explanation for this important variation of plasmatic 5-HT, depending on androgen status, is completely unknown. Given that gut enterochromaffin cells (EC1) produce most of endogenous 5-HT, one can speculate that androgens may influence the production of 5-HT by EC1 cells, pointing to a completely new physiological testicular-intestinal circuit. Since the blood–brain barrier is impermeable to 5-HT, a contribution from the central nervous system (CNS) is highly improbable^30^. However, androgens negatively regulate the production of 5-HT in the CNS, implying a similar effect to the peripheral regulation^31^. Also, platelets are unlikely to explain this observation because platelet 5-HT does not depend on castration, as previously reported^32^. Hence, further studies are necessary to elucidate TES’s role in 5-HT gut production.

In this study, a positive correlation between plasmatic and prostatic 5-HT was found. Curiously, and corroborating our findings, animals infused continuously with 5-HT exhibited higher prostatic 5-HT levels, than organs such as the liver, main vessels, or jejunum^33^. Moreover, and in agreement with our previous findings that revealed that 5-HT administration can decrease prostate growth, we now show that the modulation of plasmatic 5-HT directly determines prostatic 5-HT concentration. Overall, it may suggest that pharmacological modulation of peripheral 5-HT can be used to efficiently increase prostatic 5-HT and, in this way, inhibit prostatic growth. These findings are also consistent with a preceding study in a large cohort of human males, that established that LUTS were associated with benign prostate increase and plasmatic 5-HT decrease^10^. Furthermore, Sayed et al*.* showed that dapoxetine, a selective serotonin reuptake inhibitor, may inhibit prostatic growth significantly^23^. Consequently, low plasmatic/prostatic 5-HT levels may be a risk factor for BPH development. The normalization of plasmatic/prostatic 5-HT levels may eventually be used as a future therapeutic approach for BPH.

Lastly, our data demonstrated that treatment with different doses of TES did not induce different prostatic growth which is in agreement with the saturation model proposed by Morgentaler et al.^7^.

## Conclusions

Overall, our data revealed that mice prostate is a rich 5-HT organ and that its levels are independent of androgens. Additionally, we also showed that plasmatic 5-HT concentration increases after castration, decreases after androgen supplementation, and may positively correlate with prostatic 5-HT. We found an association between TES modulation and plasmatic and prostatic (5-HT) levels. Considering that normal prostatic growth is highly dependent on TES and 5-HT influence, it is plausible that this mechanism might be impaired in pathological prostatic growth, as, for instance, BPH. These findings favour the serotoninergic inhibitory pathway as a potential new medical therapeutic approach for BPH treatment.
